# A Model for Dental Age Verification Using Ultrastructural Imaging for Modern and Fossil Representatives of the Rhinocerotidae Family

**DOI:** 10.3390/ani11030910

**Published:** 2021-03-22

**Authors:** Edyta Pasicka, Dariusz Nowakowski, Robin Bendrey, Oleg P. Melnyk

**Affiliations:** 1 Archaeozoology Laboratory and Museum of Standards, Division of Animal Anatomy, Department of Biostructure and Animal Physiology, Faculty of Veterinary Medicine, Wrocław University of Environmental and Life Sciences, Kożuchowska 1/3, 51-631 Wrocław, Poland; 2 Department of Anthropology, Wrocław University of Environmental and Life Sciences, Kożuchowska 5, 51-631 Wrocław, Poland; dariusz.nowakowski@upwr.edu.pl; 3 School of History, Classics and Archaeology, Old Medical School, University of Edinburgh, Teviot Place, Edinburgh EH8 9AG, UK; robin.bendrey@ed.ac.uk; 4 Department of Animal Anatomy, Histology and Pathomorphology, Faculty of Veterinary Medicine, National University of Life and Environmental Sciences of Ukraine, Potekhin 16, 03041 Kyiv, Ukraine; museum@nubip.edu.ua

**Keywords:** age estimation, dentition, cement, dentine, light microscope (LM), scanning electron microscope (SEM), white rhinoceros

## Abstract

**Simple Summary:**

Determining the age of an animal based on remains, especially fossil remains, is challenging. The age of an individual can be estimated from ultrastructural analyses of teeth. Therefore, in this study, an attempt was made to establish a relationship between the age of a rhinoceros and the ultrastructure of its tooth. The subject of these examinations was a tooth that originated from a female white rhinoceros that died at Kiev Zoo. The age of the female was known. She did not give birth during her life, so pregnancy did not influence the tooth ultrastructure. For ultrastructural examination, ground sections of the tooth were obtained and slides were observed under a light microscope in white light as well as in polarized light with the use of a lambda filter. In addition, gold-coated preparations were imaged in a scanning electron microscope. Three sections were cut out of the specimen: Horizontal through the tooth crown, horizontal through the upper part of the root, and longitudinal. In subsequent stages they were investigated to see the annual growth lines of mineralized dental tissues of cement and dentine. These lines were counted from the root canal center to the cheek surface of the tooth. The most satisfactory results were obtained on the horizontal section through the upper part of the root, where distinct growth lines were observed in the dentine, and their number for both roots was consistent with the known chronological age of the animal.

**Abstract:**

The analyses were performed on a right third premolar (P_3_) of a white rhinoceros female (*Ceratotherium simum*, Burchell 1817). The specimen was born in captivity at London Zoo (Zoological Society of London), then in the 1970s transferred to Kiev Zoo (Peremohy Avenue), Ukraine, and was kept there until it died at a documented chronological age of 48 years. The female died because of its age, which indicates it was kept in good conditions adequate to the requirements of this species. Photographs and micrographs with radiological documentation were taken on the said tooth. Its structural characteristics were determined, and on the occlusal surface areas and points of anatomical constitution of its crown were identified. The tooth was also histologically evaluated via sections taken horizontally in a mesial-distal plane through the crown, horizontally in a mesial-distal plane through the coronal portion of the root, and longitudinally in a lingual-buccal plane through the crown and the root. Preparations with ground sections were made and observed in white, polarized, and reflected light. In the subsequent stage X-ray and SEM imaging has also been used, for analysis of the distribution of annual growth layers of mineralized dental tissues of cement and dentine, counted from the root canal center to the buccal surface. An attempt was also made to confirm the annual season in which the animal died, based on cement growth lines. It was observed that the growth lines were visible in all the analyzed sections, in dentine and cement. In the cement, the lines were relatively few and did not represent the attested age of the animal. The analysis of the coloration of the cement lines indicated that the animal was regularly fed a diet that was not seasonally differentiated. From the X-ray examination comes a conclusion that the animal did not suffer from periodontal diseases. Visible growth lines were observed on the dentine. On the horizontal section through the crown growth lines in the dentine were few and unclear. On the longitudinal section, both on the caudal and rostral roots, these lines were clearly visible and much more numerous than expected considering the known age of the animal, as more than 50 were counted. On horizontal sections through the upper part of both roots, distinct growth lines were observed in the dentine, and their number—48 for both roots—corresponded precisely to the age of the animal. The results of our study indicate that this method has significant potential for application to verify the age at death for modern and fossil representatives of rhinoceros.

## 1. Introduction

Animal remains on paleontological and archaeological sites are among the most numerous findings [1,2,3]. Animal material explored from these sites is, among other inputs, the source of information for interdisciplinary fields of research such as paleontology and archaeozoology, which seek answers to different questions. In archaeozoology, an important aspect is the identification of the role of animals in human socio-cultural development, and in paleontology, it is recognizing and contextualizing ancient forms of life based on fossil organisms, dated long before the appearance of man.

Commonly applied research methods, used in both fields, involve zoological and anatomical identification of the remains of different animal species [1,2,3], assessment of paleopathological changes [2,4,5,6,7,8,9,10], as well as taphonomic marks on bones, identification of sex, estimation of withers height [2,4,5,6,11,12,13], and determining the morphological type [2,4,5,6,7,14], season, and also the animal’s age at death. In fossil studies, it is important to determine the individual age of death of animals because of the utility of this information for, among other analyses, the estimation of mortality patterns (catastrophic profile and natural mortality profile). The first mortality pattern—the catastrophic profile—reflects the normal age distribution of a population; in general, the older the age group, the smaller the number of individuals. It could occur as a result of natural disasters or mass chasing of animals into traps. The natural mortality profile, on the other hand, shows a relative over-representation of young and older animals, compared to the numbers found in the populations of living species. It could occur as a result of animal death, disease, hunger, accidents, or predator hunting [15]. Animal age at death can be analyzed on the basis of both the axial skeleton and the skeleton of the extremities. This works especially well in morphologically immature animals, where the age is determined by ossification points on the bones, the degree of obliteration of the cranial sutures, and the time of teeth eruption or replacement. On the other hand, in individuals where post-cranial fusion is complete, a more precise method of age estimation is the assessment of the degree of attritional wear on teeth assessed through the shape of the occlusal surfaces [2]. One should remember that these commonly used methods of age estimation are typically based on estimates derived from present-day animals, and we must be aware when assessing the age of animals living in the past that they may be characterized by a slower rate of development. These methods are often used with an unclear degree of approximation.

According to various researchers, satisfactory results in age assessment are provided by ultrastructural analyses of dental tissues, especially cement [16,17,18,19]. In the literature, different methods for the analysis of cement growth lines can be found, as well as different techniques for the preparation of ground sections allowing for their detailed imaging [16,20,21,22,23,24,25,26]. Cement microstructure analysis can also provide information about the season in which the animal died [18,23,27]. Building on this foundation, the aim of the research presented here is to explore correlations between the ultrastructure of different dental tissues and the known individual age of a white rhinoceros and annual season of the animal’s death.

The rhinos, similarly to representatives of Pleistocene megafauna such as elephants and hippopotamuses, among others, started to become extinct in a context of Quaternary climatic changes, being replaced by herbivores better adapted to the conditions that followed. From the order Perissodactyla, still very numerously represented by rhinoceros in the Tertiary, despite the different views of researchers on this division, only four genera within three tribes of one Rhinoceratidae family with five species survived until today. In Southeast Asia, these include Sumatran *Dicerorhinus sumatrensis*, Indian *Rhinoceros unicornis,* and Javan *Rhinoceros sondaicus*; and in Sub-Saharan Africa black *Diceros bicornis* and white rhinoceros *Ceratotherium simum* [28,29,30,31,32]. The white rhinoceros (African, square-lipped) is a representative of the largest of the aforementioned species and, like the rest of the family, is almost completely wiped out [33]. Since the specimen investigated in the present study belongs to an endangered species [34,35], it is appropriate to expand the existing literature to include information on age estimation based on its dentition. The research presented can be used to analyze present-day rhinoceros specimens, but it will be primarily useful for considering the age of extinct representatives of the Rhinoceratidae family found in fossil assemblages (for instance: Woolly rhinoceros *Coelodonta antiquitatis*).

## 2. Materials and Methods

### 2.1. Study Animal and Tooth Analyzed

**Figure 1:**
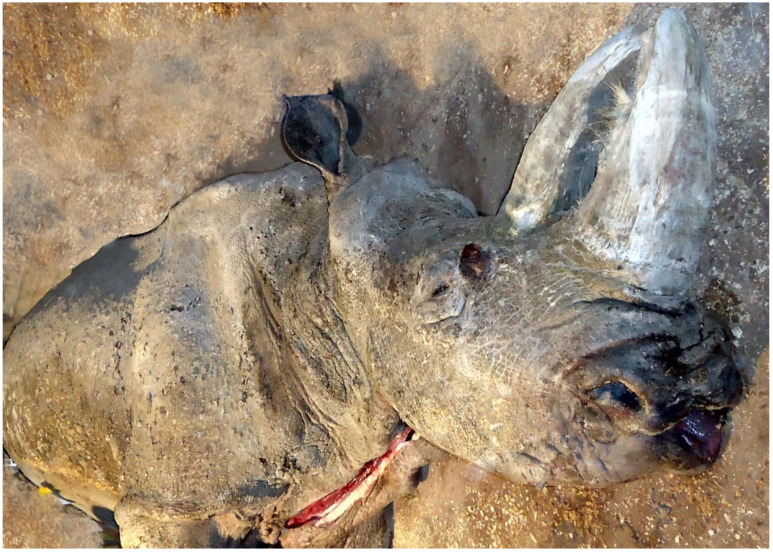
48-year-old white rhinoceros female (*Ceratotherium simum*, Burchell 1817), which died in Kiev Zoo, Ukraine.

The skeletal material selected for analysis was the right lower third premolar (P_3_) (*dens praemolaris tertius inferior dexter*) from the mandible of a white rhinoceros female (*Ceratotherium simum*, Burchell 1817). This animal died on 22 March 2016 at 48 years age, on the premises of Kiev Zoo, Ukraine. For a post-mortem diagnosis, the animal was transported to the National University of Life and Environmental Sciences of Ukraine (NULES). During the autopsy, staff of the Department of Animal Anatomy, Histology and Pathomorphology determined the animal died from natural causes resulting from its advanced age (Figure 1).

**Figure 2:**
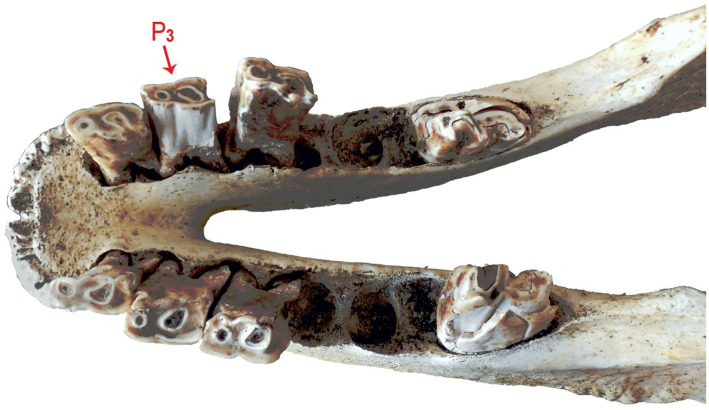
Dorsal view of the mandible of the white rhinoceros female, with the analyzed P_3_ marked.

**Figure 3:**
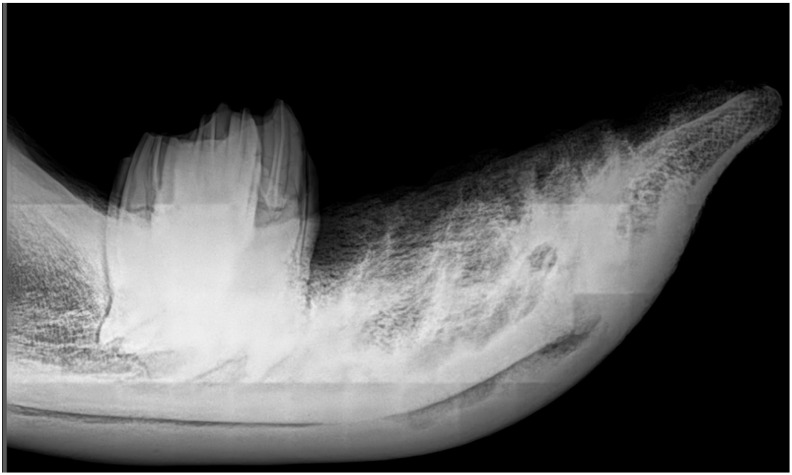
Lateral view X-ray of the mandible of the white rhinoceros female (only M_3_ teeth firmly fixed in alveoli left for imaging).

It should be mentioned that preparing animals of this size is a rather difficult task, especially when it is not just a post-mortem to determine the cause of death, but as in this case, a post-mortem with the aim of the preservation of the integrity of musculoskeletal structures for comparative anatomical studies. During this process of preparation and preservation, of the mandible *mandibula*, a photographic documentation was made (Figure 2) together with an X-ray in lateral projection (Figure 3).

**Figure 4:**
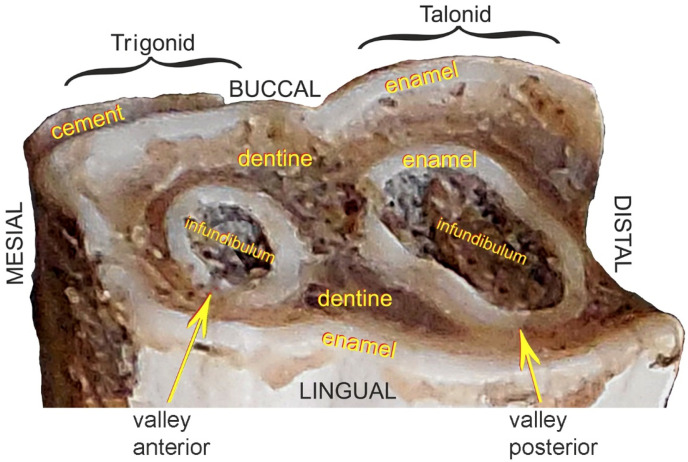
Description of anatomical structures of the third premolar (P_3_) of the white rhinoceros female.

For the examined tooth, histological characterization of its dental structures was undertaken, and on the occlusal surface areas and points of the anatomical build of the crown were identified, according to the terminology recommended by Fortelius [36], Prat [37], Antoine et al. [38], and Böhmer et al. [39] (Figure 4).

### 2.2. Specimen Preparation

The tooth was placed in acetone (CH_3_COCH_3_, p.a. grade, 100%) twice for 7 days to dehydrate it. Then the tooth was dried for 24 h and placed in methylacrylate for 7 days, which in turn was intended to thoroughly saturate the preparation with this compound. In the next stage the entire preparation was immersed in a resin consisting of methylacrylate in proportions 100 parts by weight (methyl methacrylate ≥99%, C_5_H_8_O_2_, M 100, 12 g/mol, density 0.94); polyethylene glycol 10 parts by weight (PEG 400, 99.5% purity, CAS: 25322-68-3, M 400 g/mol, C_2n_H_4n+2_O_n+1_), and 1 part by weight benzoyl peroxide solution (benzoyl peroxide 70%, remainder water, (C_6_H_5_CO)_2_O_2_). To harden the resin and simultaneously protect the tooth against crushing at further stages of the procedure, the preparation was left in the resin for 48 h at T = 25 °C and then for another 48 h at T = 35 °C [9].

### 2.3. Analyzed Tooth Sections and Type of Equipment Used

**Figure 5:**
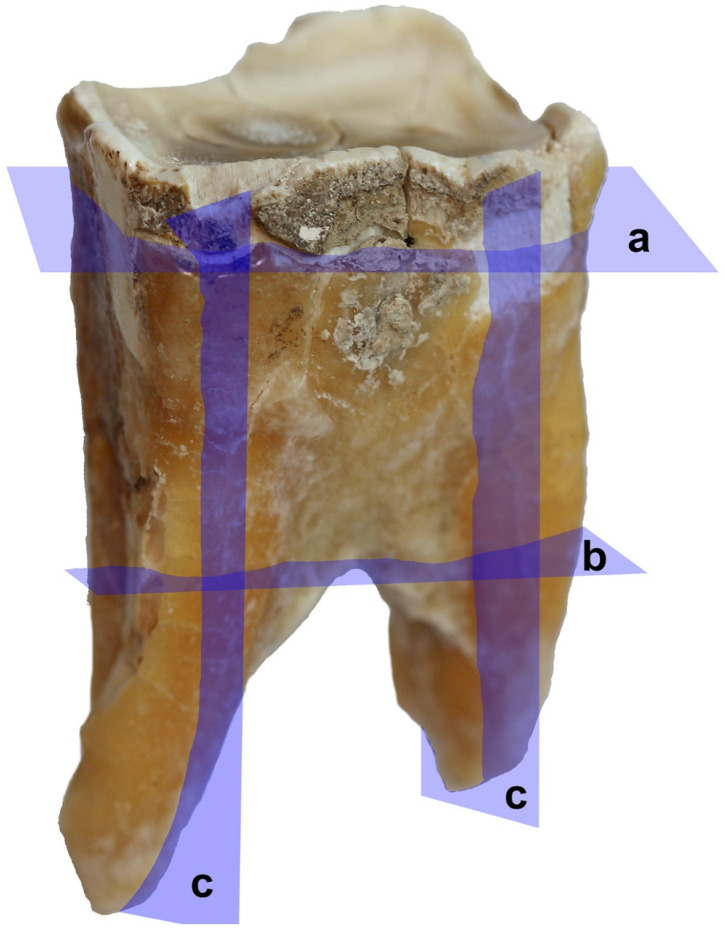
Cut sections on the third premolar (P_3_) of the white rhinoceros female: **a**: Horizontal section through the tooth crown; **b**: Horizontal (axial, transverse) sections through the upper part of the root; **c**: Longitudinal sections.

Three sections were cut out of the hardened specimen: Horizontal in a mesial-distal plane through the crown, horizontal in a mesial-distal plane through the coronal portion (upper part) of the root, and longitudinal in a lingual-buccal plane through the crown and the root (Figure 5). In the next stage, these were analyzed with the aim of identifying patterns of annual growth lines of mineralized dental tissues (cement and dentine), counted from the center of the root canal to the buccal surface of the tooth:

(a) horizontal in a mesial-distal plane through the tooth crown—the cutting plane positioned at a height of approximately 30 mm from the occlusal surface;
(b) horizontal (axial, transverse) sections in a mesial-distal plane through the coronal portion (upper part) of the root—cutting planes positioned on the rostral and caudal root, at the height of the bifurcation of the tooth roots;
(c) longitudinal sections in a lingual-buccal plane through the crown and the root—cutting planes routed through the middle of the rostral and caudal roots.

Sampled sections, 0.5 mm thick, were made using a BUEHLER^®^ IsoMet^®^ diamond low-speed saw (Lake Bluff, IL, USA). The surfaces were then sanded on a BUEHLER® MetaServ^®^ 250 (Lake Bluff, IL, USA) precision grinder-polisher using 600, 1200, and 4000 grit BUEHLER^®^ papers. Preparations created this way were then observed under a Nikon^®^ ECLIPSE 80i, Tokyo, Japan light microscope in white and polarized light with a lambda filter. Scale bars indicating magnification are added to the figures. Gold-coated preparations were observed using a Zeiss^®^ EVO LS 15 scanning electron microscope (Oberkochen, Germany).

### 2.4. Annual Season Analysis Based on Cement Lines

**Figure 6:**
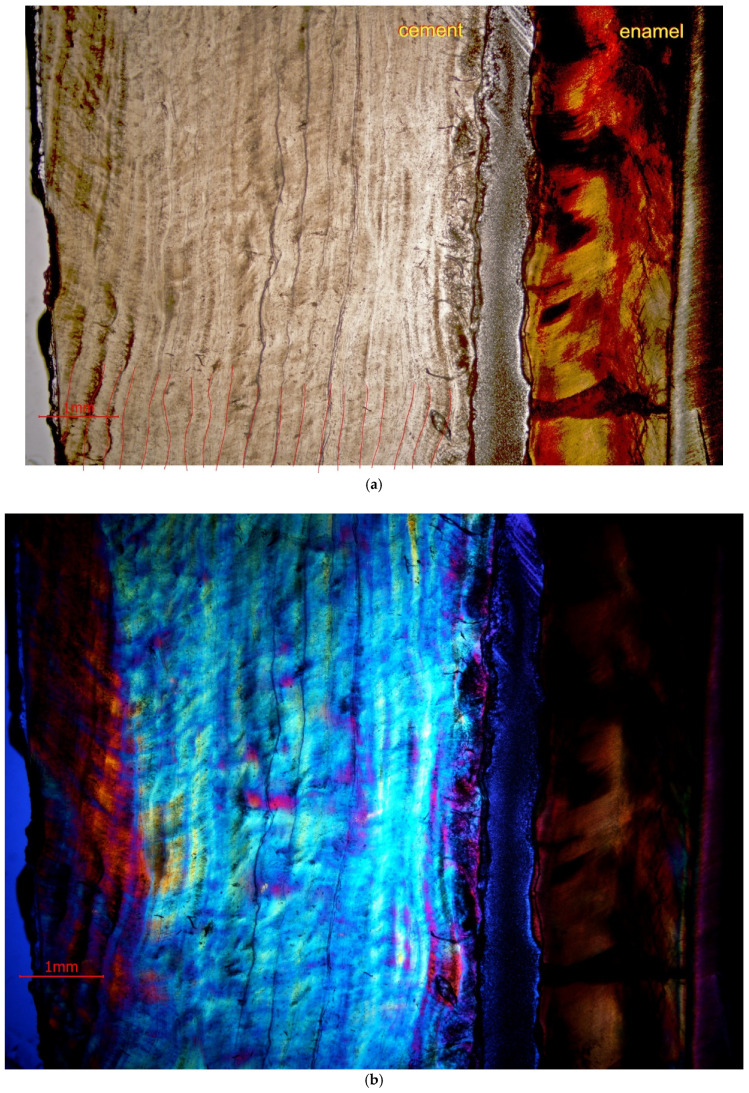
(**a**). Cement growth lines on the third premolar (P_3_) of the white rhinoceros female. The part of the tooth analyzed here is indicated by a red arrow in Figure 8. (**b**). Cement lines on the third premolar (P_3_) of the white rhinoceros female in polarized light. The part of the tooth analyzed here is indicated by a red arrow in Figure 8.

In addition to analyzing the number of growth lines within mineralized tissues (cement and dentine), an attempt was made to determine the annual season at which the animal died, based on the cement growth lines. These lines were observed on flat scans in reflected light at a 9600 dpi resolution and in light microscopy Figure 6a,b.

## 3. Results

### 3.1. Analysis of Tooth Sections

**Figure 7:**
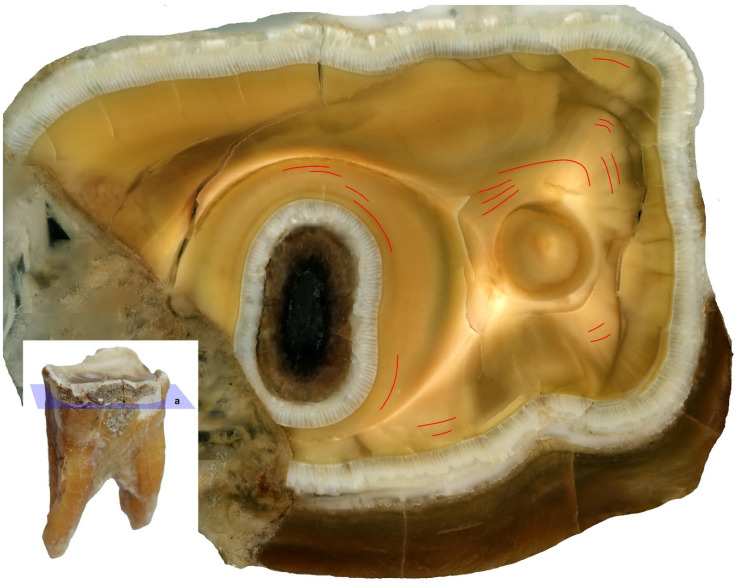
Dentine growth lines on the horizontal section through the tooth crown on the third premolar (P_3_) of the white rhinoceros female, **a**: Horizontal section through the tooth crown.

Growth layers are formed and are independently observed in the spatial structure of dentine, from canal to tooth margin. In our research we observed that on the same specimen, on the designated sectioned planes, the lines were not equally visible in terms of their number and clarity of observation. Since these are the layers of annual growth, we were interested in their largest possible number on each of the studied sections. It was found, on the basis of observations of growth layers, that they were visible on all analyzed sections, on dentine and cement. On cement, approximately 19 layers were observed (Figure 6a), while the highest number of growth lines were observed on dentine. On the horizontal section of the tooth crown plane, these dentine growth lines were few and often unclear (Figure 7).

**Figure 8:**
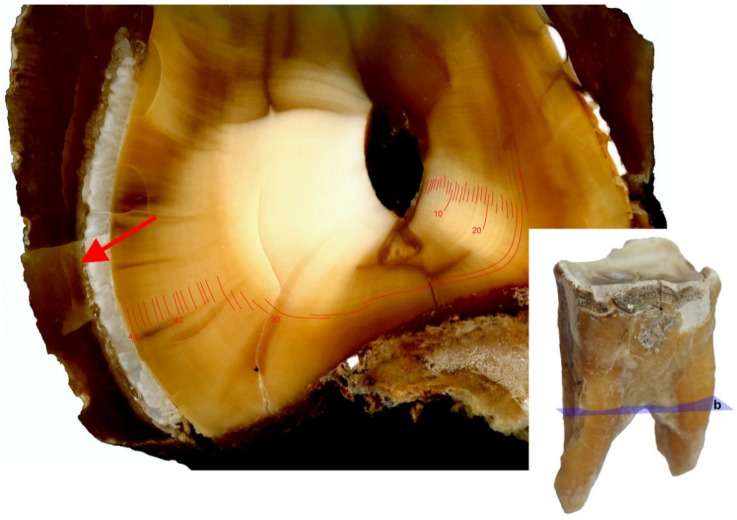
Dentine growth lines on horizontal (axial, transverse) sections of the upper part of the caudal root, on the third premolar (P_3_) of the white rhinoceros female, **b**: Horizontal (axial, transverse) sections through the upper part of the root. The cement area analyzed in Figure 6a,b indicated with red arrow.

On horizontal sections through the coronal portion (upper part) of the root clear growth layers were observed in the dentine. Further, the number of lines recorded in both roots (48) matched the age of the animal (Figure 8).

**Figure 9:**
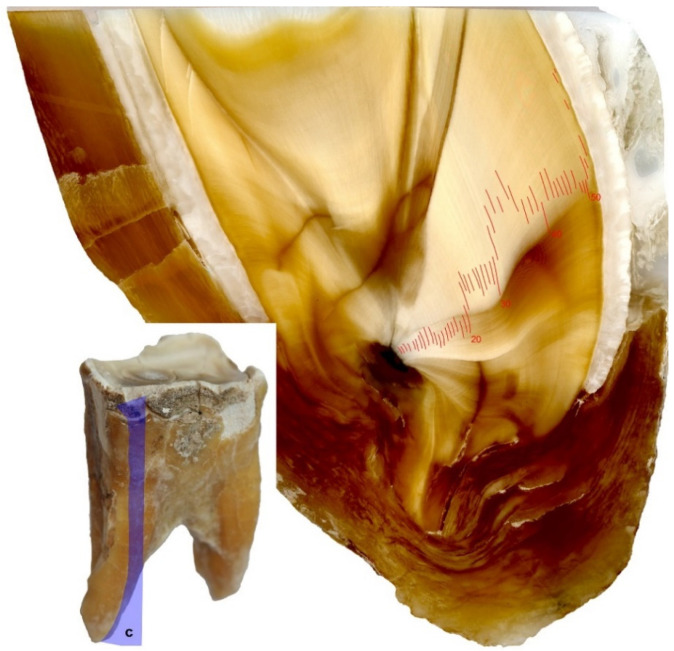
Dentine growth lines on longitudinal section, on the third premolar (P_3_) of the white rhinoceros female, **c**: Longitudinal section.

On the longitudinal sections, both on the caudal and rostral root, these layers, although clearly visible, significantly exceeded the expected value due to the known age of the animal because more than 50 were counted (Figure 9).

### 3.2. Analysis of Seasonality Based on Cement Lines

The coloration of the annual growth layers on cement is related to seasonality, and their thickness, according to a number of authors, is related to the biological condition of the animals. In the studied specimen no differences in color and thickness of the cement layers were observed (Figure 6a,b).

### 3.3. X-ray Imaging of the Mandible

In our study, no inflammatory changes in the structure of bone tissue or the structure of the tooth were visible in the radiological image, within the alveoli, alveolar processes, and body of the mandible (see Figure 2 and Figure 3).

## 4. Discussion

Each species of mammals has a specific type of dentition, determining the number and shape of teeth. The cheek teeth of this group originate from two primal types: In the upper arcade from the tribosphenic tooth, and in the lower arch from the tuberculosectorial one, which has a trigonid in the mesial part and a talonid in the caudal [37]. An example of the latter is the evolutionary transformed cheek tooth of rhinoceros (see Figure 4).

**Figure 10:**
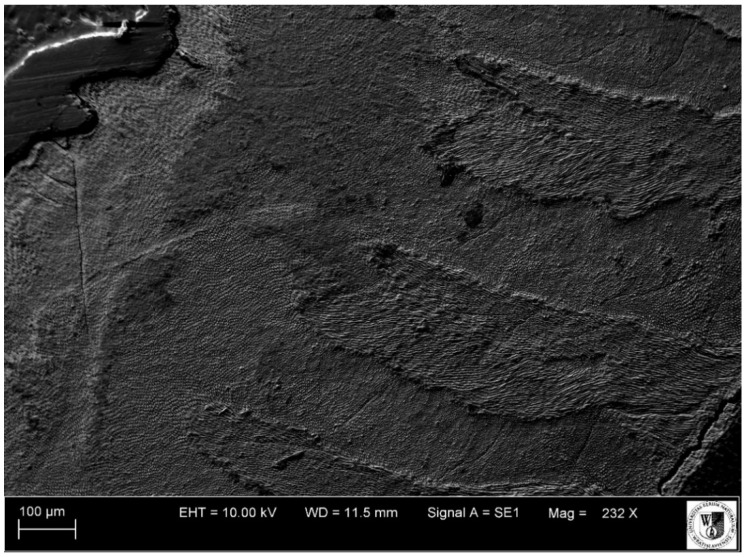
SEM micrograph of enamel (horizontal section through the crown) on the third premolar (P_3_) of the white rhinoceros female. EHT: Electron high tension voltage; WD: Working distance between the sample surface and the low portion of the lens; Signal A = SE1: Secondary electrons emitted from the top of the sample surface resulting from interaction with primary beam.

**Figure 11:**
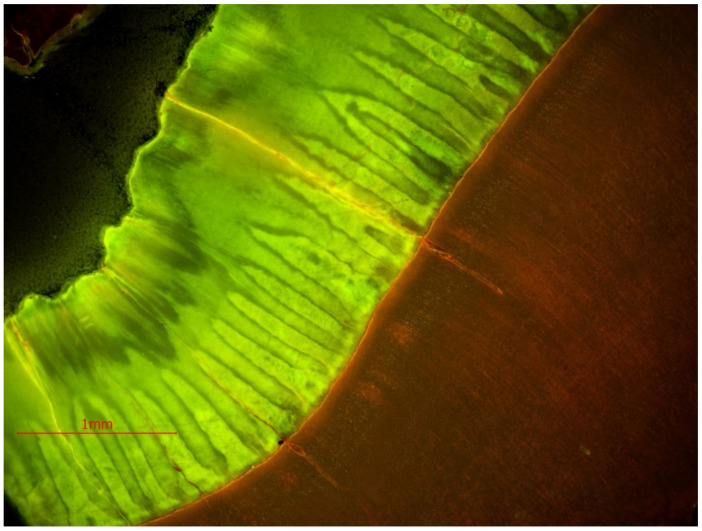
Image of enamel (horizontal section through the crown) on the third premolar (P_3_) of the white rhinoceros female under a light microscope using a B20UV filter.

**Figure 12:**
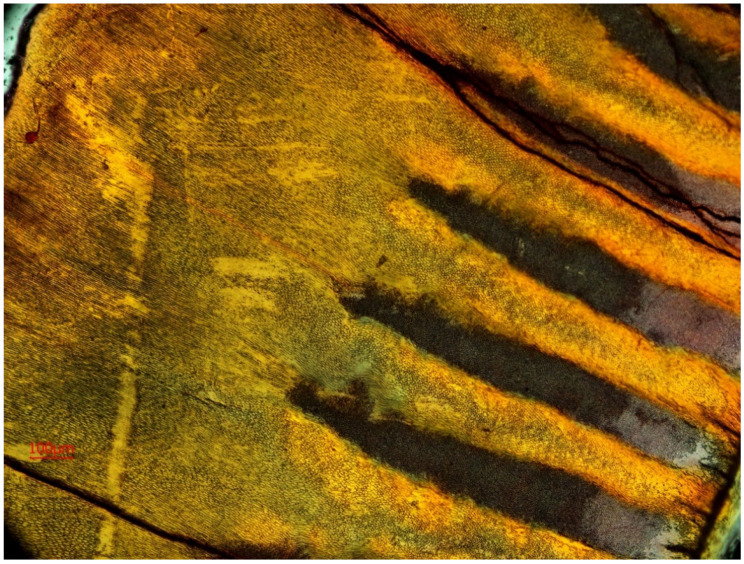
Image of enamel (horizontal section through the crown) on the third premolar (P_3_) of the white rhinoceros female under light microscope using polarization.

**Figure 13:**
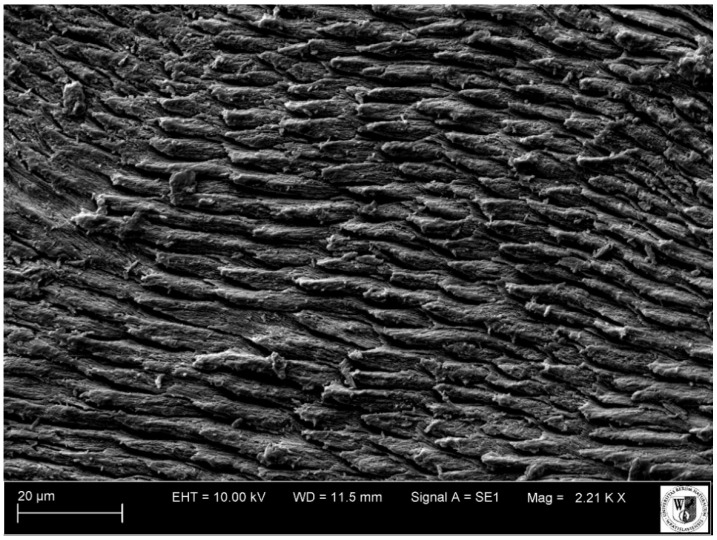
SEM micrograph of enamel (horizontal section through the crown), view of prisms on the P_3_ mandible tooth of the white rhinoceros female.

**Figure 14:**
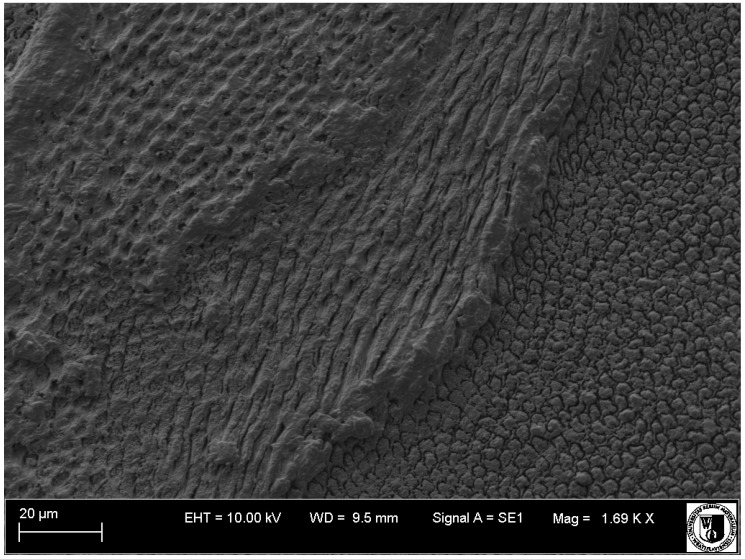
SEM micrograph of enamel (horizontal section through the crown), view of prisms—oblique (central) and vertical (right) on the third premolar (P_3_) of the white rhinoceros female.

Despite their highly specialized structure, mammalian teeth have common structural features [40,41,42]: The crown corona dentis and the root *radix dentis*. Depending on whether we deal with a low-crowned brachydont tooth or a high-crowned hypsodont tooth, we can respectively distinguish the neck *collum dentis* or the long body of tooth *corpus dentis*, which compensates for the attrition of the tooth by gradual eruption from the alveolus. Each tooth is built up of three mineralized tissues: cement *cementum*, dentine *dentinum* and enamel *enamelum*. In addition, depending on the enamel layout, we distinguish between brachydont and hypsodont teeth, which include cheek teeth of herbivorous animals, including rhinos. In the latter, arrangement of the harder tissue—enamel under the more plastic cement—produces an increased resistance to abrasive food, creating an occlusal surface with numerous lofodontic-type folds and crests. This type is characterized by an uneven tooth grinding surface caused by slower wear of the enamel laminas in relation to the layers between them: Outer-cement and inner-dentine [36,43]. This feature predisposes these lofodontic teeth to grinding of a diet composed of hard vegetation (Figure 10, Figure 11, Figure 12, Figure 13 and Figure 14).

In the chemical composition of the tooth tissues, inorganic substances predominate, especially hydroxyapatite crystals, which give hardness to these tissues. The hardest tissue is enamel, built of prisms and interprismatic substance, produced by ameloblasts [44]. Enamel is a tissue that does not regenerate. Dentine and cement, unlike enamel, can be produced over a lifetime, creating growth lines by deposition. The dentine is produced by odontoblasts up to the senile age of an animal. Odontoblasts, by producing secondary dentine, fill the tooth cavity and replace the pulp with it. In dogs, the process of reducing the dental cavity through filling it with secondary dentine may be an indicator of the individual’s age [40]. Cement, the structure of which resembles bone tissue, is produced by cementoblasts. It is the least hard of the dental tissues and is more resistant to compression compared to dentine and enamel [40,41]. Cement can be deposited continuously throughout life; however, seasonal differences in the degree of deposition of this tissue result in layered lines, which are laid down like annual growth rings in trees [23,27]. According to the research by Ruscillo [27], thinner and darker lines are the growth period of cement in winter, while lighter and thicker-growth period in spring and summer. In wild animals, especially those from higher latitudes, where the climate is more strongly seasonal, this clear division between the rings is maintained. In domesticated animals and those from areas with less extreme seasonality, the increments of cement layers may not create the aforementioned characteristic image. Therefore, if no differences in color and thickness of layers of cement deposited in the examined specimen were recognized, it is likely that the animal was fed regularly, with an undifferentiated diet during the year.

By analyzing the image of the path of the cement growth lines under the light microscope, on all the planes under consideration, it was possible to determine their minimum number. In our study, about 19 lines on cement were observed. Such a small number of them—in relation to the 48 years of life of the rhinoceros in question—may be due to the already very advanced age of the animal, as the rate of cement deposition changes with the passing of years. The older the individual is, the more densely arranged the increments are and, thus, more difficult to interpret [22]. Apart from that, our examination shows that an overview image of the cement lines on the analyzed planes is more readable under a light microscope than in a scanning electron microscope, which is dedicated to detailed imaging at very high magnifications.

Age estimation based on cement ultrastructure has already been addressed for various animal species in multiple studies, for example those belonging to the predatory order Carnivora, and bears in particular. In their fossil representatives—cave bears *Ursus ingressus* [45] and *Ursus spelaeus* [22]—first molars, but also canine teeth, were analyzed in this aspect [18]. In turn, the first premolar was recommended for age analyses in black bear *Ursus americanus* [46]. First molars were preferred for ultrastructural analyses also in the case of representatives of the order of Perissodactyla in extinct rhinoceros *Stephanorinus kirchbergensis* [47] as well as in the modern white rhinoceros *Ceratotherium simum* [48]; in the case of fossil and present-day *Equus caballus* representatives, different cheek teeth (first and second molars, third and fourth premolars) were preferred [49]. In *Bos taurus* cattle, first molar was examined in this respect [50], also in bison *Bison bison* [51], and in another representative of the Artiodactyla order—sheep *Ovis aries*—incisors were targeted [52].

According to the observations of various researchers, the kind of teeth picked for ultrastructural imaging is not negligible. In the case of teeth that erupt almost a year later than the others (this relates to the last cheek teeth and the canines), an additional year should be added when quantifying the cement growth lines. Moreover, the last molars are not recommended for the analyses of cement growth lines due to occurrences of anomalies and irregularities in the cement layering [18,23,53], nor are the incisors due to more limited expression of this dental tissue compared to cheek teeth [54].

In this study, the analyses performed on dentine, on three of the analyzed sections, provided interesting observations regarding the growth lines of this tissue. The most satisfying results were obtained on horizontal sections of the upper part of the root, where clear growth lines were observed on dentine and their number for both roots was consistent with the age of the animal (see Figure 8).

The dentine structure was also investigated with attention to its growth lines, but mainly in the context of daily incremental lines known as von Ebner lines, during the period of teeth formation, especially mammalian teeth [55,56,57,58,59]. Waugh et al. [26], in their research on growth layers in the dentine of beluga, interpreted short-period incremental lines (von Ebner lines) that are assumed to represent daily pulses of dentine mineralization. Beluga teeth have an open pulp cavity, which fills with dentine throughout the whole life of the animal creating the growth layers. Between the growth layers created in this fashion, further incremental lines are deposited daily. Based on research by Waugh et al. [26], it takes about 365 days for short-period incremental lines to fill the space between two growth layers. It has thus been demonstrated that these growth layers are deposited annually, and the number of which are commonly used as an indicator of age in marine mammals.

The method of age verification based on the lines of growth on horizontal sections of the upper part of the root’s dentine, developed in this study, give promising and verifiable results for modern material for which the age at death was known. This method needs to be tested on dentition of present-day animals—domestic as well as wild. In addition, the method should be applied to both archaeozoological and paleontological materials. Fossil assemblages, even those with teeth discovered, are often subject to a complex of taphonomic factors, relating to depositional and environmental parameters. It is obvious that the best for ultrastructural research would be the remains originally not damaged by factors weakening their structure such as soil hydration, temperature spikes, soil type, and its grain size, as well as factors related to human activity. However, most fossil findings are exposed to this type of taphonomic factors, due to their presence in the earth during a period that can last up to several million years. Additionally, during preparation of ground sections, the teeth are subjected to mechanical factors related to their processing. Despite being embedded in substances that protect and solidify them (resins), teeth have natural cracks that can affect analysis of their surface. In herbivorous animals, cement, as the outer dental tissue, both on the crown and the root of the tooth, is most exposed to various types of damage. Consequently, growth lines counted on this tissue in the case of fossil material that is destroyed, damaged, or comes from a very old specimen (as it was in our case) may not accurately show the age of the animal. We propose that for this reason, age analysis in herbivores on the centrally placed and thus protected tissue of dentine is beneficial, in relation to the externally placed cement and enamel.

From the X-ray and inspection of the mandible alone (see Figure 2 and Figure 3), we conclude that the animal did not suffer from periodontal diseases *paradontitis*—inflammation of the entire apparatus (*alveolitis* and *periostitis*)—the apparatus that kept the rhinoceros’ teeth in their physiological position in supravital conditions. Such an inflammation would leave a permanent mark on the mandible, as it is an irreversible process involving bone tissues, and in its acute form also involves tooth cavities. The secondary inflammation of the pulp itself is manifested by its pyogenic inflammation, caused by bacteria penetrating through foramen apicale of the dental roots. This, in turn, can lead to periorbital changes, formation of purulent fistulas destroying soft tissues and skin, and consequently opening on the inside of a mandible’s margin [7,42,60], which is not present in this specimen.

## 5. Conclusions

In this paper, we demonstrate that counting dentine growth layers on horizontal sections through the coronal portion (upper part) of the root of the rhinoceros allowed us to precisely confirm the known age for the specimen. This suggests that this method can be used for the interpretation of age in other rhinos, but potentially also in other present-day domestic and wild-living animals. First and foremost, however, this method is appropriate for application to fossil collections. The method gave positive and expected results for the premolar tooth, its usefulness should now be tested on other teeth. Under the light microscope, the analysis of cement growth lines is possible on all the investigated planes, but readability of the lines depends on the animal’s age. In our case, light microscope imaging produces better results than SEM imaging. Based on the experience from processing preparations for a modern animal, additional practical conclusions arise. Namely, when using the same methods for fossil materials, one must remember that these materials are much more fragile and susceptible to crushing and damage during preparation of the ground sections, and also the images from a fossil preparation prepared in such a way may hinder interpretation of the results. Moreover, embedding the slide in a synthetic resin, in order to protect the tooth from mechanical damages during processing, is an irreversible process, and this fact should also be taken into account when planning the selection of material for research.

## Data Availability

The data presented in this study are available on request from the corresponding author.
